# Surviving the glocal ecosystem: Academic dissonance, cultural adaptation, and coping among international medical students in the Caribbean

**DOI:** 10.3389/fmed.2026.1937943

**Published:** 2026-10-26

**Authors:** Mohamed M. Al-Eraky, Sofiia Belogubova, Mikhail Kondratiuk, David Taylor

**Affiliations:** 1 Department of Medical Education, Arabian Gulf University (AGU), Manama, Bahrain; 2 Department of Medical Education, American Canadian School of Medicine, Portsmouth, Dominica; 3 Department of Medical Education, Gulf Medical University (GMU), Ajman, United Arab Emirates

**Keywords:** cross-cultural adaptation, international medical education, phenomenological study, student mobility, the Caribbean, cultural challenges, coping strategies, academic dissonance

## Abstract

**Introduction:**

The globalization of medical education has increased international student mobility, with many students choosing to study abroad to gain exposure to different healthcare systems and cultural environments. Despite this global trend, limited research exists on the experiences of international students attending newly established medical schools in developing regions. This study aims to explore the decision-making process, motivations, challenges, and adaptation strategies of international medical students at a brand-new medical school founded in 2023 in the Caribbean.

**Methods:**

A qualitative, descriptive phenomenological approach was employed to explore students’ lived experiences. Semi-structured interviews were conducted with 12 international students of various backgrounds and demographics. The interview questions focused on decision-making, perceived benefits, academic and cultural challenges, and coping strategies. Thematic analysis was used to identify key patterns and themes.

**Results:**

Students were primarily motivated by the ability to begin their medical education without delays, citing flexible admissions, immediate program availability, and small class sizes. Challenges included academic rigor, adjusting to the flipped classroom model, cultural differences, homesickness, limited island resources, and concerns about the school's newness. Adaptation strategies included personalized study methods, mindfulness, physical activity, social support networks, and environmental resourcefulness.

**Discussion:**

International students chose Caribbean medical schools for accessible admissions, personalized education, and career alignment despite financial, social, and reputational challenges. They faced academic, cultural, and emotional pressures but used resilience and adaptive coping, achieving partial integration and academic progress, consistent with theoretical frameworks. These findings highlight the need for a comprehensive student support programme addressing academic, cultural, emotional, logistical, and reputational needs to promote well-being, retention, and successful adaptation.

## Introduction

Globalization of medical education has increased student mobility, with 11% of mobile students enrolled in health and welfare in 2022 (1, 2). This trend is driven by the desire for exposure to diverse healthcare systems and competitive admissions at home (3, 4). While offering unique educational opportunities, international medical students face multiple challenges, which can be categorized into two dimensions: personal and system-wise challenges. The personal challenges are related to language barriers, cultural adaptation, and financial constraints, and limited coping abilities, which may affect their academic performance and well-being (5, 6). The systemic challenges are related to medical schools which often lack adequate support, hindering integration and potentially lowering academic outcomes and quality of life (7, 8).

While motivations and challenges of international medical students are well-studied, their adaptation processes, especially in newly established medical schools in developing regions like the Caribbean, remain underexplored (4, 9, 10). Caribbean schools are gaining popularity among American and Canadian students, with scarce literature addressing their potential and limitations (4, 11). Understanding student adaptation in this context is essential for improving support systems, fostering academic success, personal growth, and resilience, and promoting integration into a culturally competent medical workforce (12).

### Driving factors to study medicine abroad

Analysing the driving factors that contribute to students' decisions to pursue higher education abroad is imperative to understanding the context of studying medicine away from home. The push-pull theory can be used to explain migration patterns by indicating two types of factors: the *push factors*, which drive individuals to leave their home country, and the *pull factors,* which attract them to new destinations. A modified push-pull theory, incorporating push, pull, and conditioning factors, offers a comprehensive view of the motivations for pursuing higher education abroad (13). With respect to the context of international medical education, the *Push* factors include personal goals such as enhancing skills, gaining financial stability, and personal growth, while improving language skills is also a common driver (9, 13–19). The motivations of medical students also include *preservation goals*, focused on escaping negative conditions in their home countries. Students who are driven by preservation goals tend to face greater adaptation challenges and demonstrate lower well-being (15, 16). The *Pull* factors focus on the host country's reputation, safety, cost of living, and the academic institution's standing (9, 18, 20, 21). Economic feasibility, including affordable tuition and scholarships, is a key consideration (14, 18, 19, 22). Conditioning factors like visa processes, financial resources, and support networks either help or hinder the decision to study abroad (18, 19, 22, 23).

The motivations of medical students often include a desire to travel to experience different cultures and to gain exposure to new healthcare systems, which enhances both personal and professional growth. Influences from faculty, family, and the availability of bursaries or scholarships are key factors in supporting their decision to make such a move to study medicine abroad (3, 24). However, barriers such as high costs, language challenges, and emotional strain from being away from home can impact their choices, with students generally favouring destinations where language and cultural barriers are minimal (3). Many international students are drawn to developed countries for better working conditions, higher living standards, and legal protections, while in places like Canada, the competitive admissions process pushes students to seek alternatives abroad, including direct-entry medical programs (4, 11, 25). Caribbean medical schools have a special preference for American and Canadian students due to less stringent admission requirements and curricula modelled after U.S. programs, facilitating a pathway to practice medicine back in their home countries (4).

### Challenges faced by international medical students

International medical students face several academic, social, cultural, and financial challenges while adapting to new environments. Academically, students struggle with different teaching methods, assessment standards, and healthcare systems, alongside language barriers that hinder performance and communication in clinical settings (5–7, 12). Socially, many experience isolation, homesickness, and discrimination, often exacerbated by language and cultural differences, which affect their mental health and relationships with peers (6, 8–10, 26). Cultural adaptation challenges, including difficulties with local language proficiency, medical jargon, and adjusting to different social norms and educational systems, significantly hinder international students' academic performance, social integration, overall adjustment and mental health (5, 7–9, 27–32). Financial constraints, such as high tuition fees and living costs, add stress, especially when students must support themselves during their studies (5, 9, 26, 31, 32). The COVID-19 pandemic intensified these issues, further isolating students and limiting access to academic resources (26, 33). Additionally, external factors like political instability or war in home countries can disrupt their education and well-being (28, 34).

### Theoretical framework of the study

Due to limited literature on adaptation strategies of international medical students, understanding these experiences requires a comprehensive analysis of various theoretical frameworks to guide the approach of the study and the interpretation of its findings.

*Cultural Adaptation Theories*

*Berry's acculturation model, Kim's integrative communication theory, and Bennett's DMIS all emphasize that successful cultural adaptation is multi-dimensional and hinges on integrating one's cultural identity with the host culture (35–37). Berry identifies four strategies—assimilation, separation, integration, and marginalization—with integration being the most adaptive, as it balances cultural retention with host engagement (38). Adaptation outcomes are influenced by host society attitudes, with inclusive environments fostering better adjustment across psychological, sociocultural, and academic domains (39, 40). Kim adds that adaptation is a cyclical process of stress, adjustment, and growth, facilitated by communication skills and resilience (36, 40). Similarly, Bennett's Developmental Model of Intercultural Sensitivity (DMIS) presents adaptation as a continuum from ethno-centrism to ethno-relativism, where students develop cultural competence through increasing openness to difference (37).*

*Educational Integration Models*

*Tinto's theory highlights that academic and social integration are key to student success; international students who struggle due to language, cultural gaps, or external pressures face higher dropout risks (41, 42). Schlossberg's Transition Theory uses the “4 S's” (Situation, Self, Support, Strategies) to explain how students navigate transitions, showing that strong support networks and effective coping enable better adjustment (43, 44). Both models call for combining institutional support with personal resilience to foster well-being and academic achievement.*

This study aims to understand the decision-making process behind studying medicine abroad, and to explore the perceptions and lived experiences of international medical students on the challenges they face, their coping strategies, and lessons learned from their adaptation experiences in the Caribbean. The research questions can be listed as follows:

1. What are the personal, professional, and cultural variables that contribute to the decision to study medicine in the Caribbean?
2. What are the challenges, benefits, and adaptation strategies experienced by international medical students?
3. How to use the study findings to provide tips to facilitate the transition and adaptation of international students?

## Methodology

### Context

The study was conducted from February to October 2024 at the recently established American Canadian School of Medicine (ACSOM) in the Caribbean, which admitted its first cohort in 2023. ACSOM offers an organ-system-based curriculum with flipped classrooms and small-group learning. Its student body is predominantly international, and faculty come from diverse countries, creating a multicultural environment. The school's small size and low student-to-faculty ratio provide a personalized educational experience. Most students relocate for education, bringing diverse backgrounds and motivations, making ACSOM an ideal setting to explore the challenges and adaptations they face and inform strategies for student integration and academic success. Then students are usually relocated to join residency programs in the USA or Canada, which makes ACSOM a typical *glocal ecosystem*.

### Ethics approval and consent to participate

The study received ethical approval from the Institutional Review Board of Gulf Medical University (GMU), with the reference number IRB-COM-STD-112-APR-2024.

All participants were informed about the study's purpose, procedures, and how their data would be used. Written informed consent was obtained before the interviews were conducted. Participants' identities were known to the research team during recruitment and data collection but were kept confidential throughout the research process. Identifying information was not included in the reporting of the findings, and study data were securely stored on password-protected servers accessible only to authorized research personnel.

### Study design

This study adopts a descriptive phenomenological approach to capture the lived experiences of international medical students at ACSOM. Phenomenology is centred on understanding individuals' subjective experiences and is well-suited to studying complex phenomena such as cultural adaptation and decision-making in a new educational context (45–47). Phenomenology is about documenting how individuals experience and make sense of the world around them. We went beyond the facts to explore the emotions and perceptions of international medical students studying in the Caribbean. The hidden aim of the study is to amplify the voices of individual medical students and explore commonalities and differences among them in interpreting the drivers, benefits, and challenges they experience.

### Data collection tool

Data was collected using a semi-structured interview protocol, which included 15 open-ended questions in five categories, designed to explore the following aspect: (1) the decision-making process for studying medicine abroad, (2) challenges encountered by the international medical students, (3) adaptation strategies, (4) lessons learned from their experiences and (5) tips to be shared with peers or potential international students (Table 1).

**Table 1:** The interview protocol used with the international medical students, including 15 questions in five categories.

Category	Interview question
Background	1. Tell me about yourself, background, experience and country of residence. 2. What is your educational experience?
Decision-making process	3. Why did you move here? 4. Are you happy here? Or do you regret this move? Why? 5. If you were the parent, would you do the same for your (future) kids? Why? Why not? 6. If you were not able to come here, how do you see your life and career? 7. If you had a chance to study medicine in the USA, in Europe, or in Canada, would you still consider moving here?
Challenges and Adaptation	8. After moving here, did you encounter any challenges in studying medicine? Like what? 9. How did you deal with these challenges? 10. How would you describe the environment on campus with respect to peers, teachers and the system here? 11. To what extent do you feel that the system here is different from your home country? 12. How is it different from the system in the future country of residency and work?
Benefits/opportunities	13. What are the benefits you gained from coming here? 14. What do you plan to do next, after graduation from here?
Recommendations	15. If one of your friends is planning to move here next year, what advice would you wish to give him/her?

The interview guide was developed based on previous research, peer-reviewed by experts in the field, and refined through pilot interviews (45–47). This tool was deemed suitable for capturing the complexities of the students' experiences while allowing flexibility for unexpected insights.

### Sampling strategy

At the time of recruitment, there were 14 international students who met the inclusion criteria, and all 14 were invited to participate. Twelve agreed to participate, while two did not respond; therefore, all eligible students who agreed to participate were interviewed. The study included a purposive sample of 12 international medical students enrolled at ACSOM. The inclusion criteria required participants to be non-local students who had moved to the Commonwealth of Dominica specifically for educational purposes. The sample consisted of seven male students and five female students, coming from the USA (*n* = 10), Germany (*n* = 1), and Puerto Rico (*n* = 1), all in their first or second semester of education. The demographic diversity of participants is expected to offer a range of perspectives, reflecting the multinational body of students at the ACSOM.

### Recruitment process

At the time of recruitment, ACSOM had a total of 14 enrolled students, comprising 8 students in the first cohort and 6 students in the second cohort. Thus, all eligible students were in the first year of their medical education. Participation was offered to all 14 eligible students. Of these, 12 students agreed to participate, while 2 students did not respond to the invitation; no students who responded to the invitation declined participation. The two pilot interviews were conducted with participants from this group and were included in the final dataset and analysis. The pilot interviews were used to refine the interview protocol, and no substantive changes that would preclude their inclusion in the final analysis were made following the pilot phase.

### Participants

Of the 12 participants, 10 were residing in the United States prior to enrollment, while 1 resided in Germany and in Puerto Rico. However, according to participants’ reported countries of origin, only 6 of 12 participants (50.0%) originated from the United States. The remaining 6 participants (50.0%) originated from the United Kingdom (*n* = 1), Kenya (*n* = 1), Ghana (*n* = 1), Puerto Rico (*n* = 1), Germany (*n* = 1), and Liberia (*n* = 1). Thus, although most participants had been residing in the United States before relocating to Dominica, half of the sample reported countries of origin outside the United States, reflecting a broader range of national and cultural backgrounds.

### Interview process

The interviews were conducted in English by two faculty members who had received training in qualitative research methods. Interviews were conducted individually in a private and comfortable office setting, with only the interviewer and participant present, and lasted approximately 30–80 min. All interviews were audio-recorded with participants' consent. The interviewers also maintained field notes and reflexive notes to document relevant observations and reflections during the data collection and analytical processes. The interviews were transcribed verbatim, and one member of the research team reviewed the transcripts against the original audio recordings to verify transcription accuracy before analysis.

## Data collection

Potential participants received a questionnaire, information sheet, and consent form. Twelve students agreed, and in-depth, in-person interviews were conducted in the period from April to May 2024. Two pilot interviews preceded the main study to refine questions. All interviews were audio-recorded with consent and transcribed verbatim by a professional service via NVivo to ensure accuracy and consistency. All interview recordings and transcripts were securely stored on password-protected servers, accessible only to authorized research personnel.

### The data analysis process

Thematic analysis was employed to analyse the data, following an inductive approach consistent with descriptive phenomenology (48). The researcher conducted a line-by-line review of the transcripts to identify codes, memos, and key themes, allowing for a detailed exploration of the participants' experiences. Two members of the research team independently reviewed the transcripts and developed initial codes and themes. The researchers subsequently compared their coding and preliminary thematic interpretations. Areas of convergence and divergence were discussed, related codes and themes were consolidated and refined, and differences in interpretation were resolved through discussion until agreement was reached on the final thematic structure. Field and reflexive notes were considered during this iterative process. During analysis, the research team observed that the final interviews did not generate additional major themes; however, recruitment was not terminated on the basis of saturation.

### Bracketing note

The interviewers acknowledged the concept of bracketing that is known for phenomenological studies. Despite living in the same context, they tried their best to set aside their assumptions, prior experience and perceptions. It was not an easy task, given that two of the researchers had themselves made a similar decision to move and practice medical education, as teachers, in the Caribbean a few years ago. Yet, the interviewers reminded themselves in every encounter to focus on the participants' perspectives and ensure that their personal assumptions and perceptions do not direct or moderate the participants' flow of ideas or interfere with the analysis.

### Reflexivity statement

The authors acknowledge their lived subjective stance that contributed to the study design, drafting the interview protocol, and conducting the interviews. Two of the co-authors (SB and MK), who are living in the Caribbean, were heavily engaged in the data collection. The other two authors are more senior fellows with experience of publishing qualitative studies in the same domain. As they are living and working away from the local context, in Bahrain (MAE) and in the United Kingdom (DT), they offered an outsider perspective on data interpretation, thematic analysis, and fine-tuning the final report of the study.

### Positionality statement

Given the small size of the newly established institution, two members of the research team were involved in educational, student-support, and/or administrative activities within the school. We recognize that these existing relationships could potentially introduce power imbalances, socially desirable responses, or reluctance to express critical views. To mitigate this risk during data collection, participants were interviewed by a member of the research team who was not directly involved in teaching or assessing that particular student. Participation was voluntary, and confidentiality of participants' responses was maintained. We have also acknowledged that, despite the measures taken to reduce these influences, they cannot be entirely excluded and should be considered when interpreting the findings.

### Intersubjectivity

The authors acknowledge the concept of intersubjectivity, as lived experience of participants is influenced by their interaction with each other, as members of the same cohort of junior medical students. Some of their shared motives, benefits and challenges to studying medicine abroad is moderated by their social interaction within the Caribbean context. This might be a strength of the study and support the interpretation of data and the commonalities of the themes that have emerged from their responses to the interview questions.

## Results

The study included 12 medical students from diverse backgrounds. The socio-demographic characteristics of participants are presented in Table 2.

**Table 2:** Socio-demographic characteristics of participants.

N	Age	Gender	Country of origin	Country of residence	Country/countries of temporary residence
1.	37	M	United Kingdom (England)	USA, New Jersey	Greece, USA, Wyoming
2.	25	F	Kenya	USA, Miami	USA, New York
3.	30	F	Ghana	USA, North Carolina	USA, Washington, USA, Philadelphia
4.	26	F	USA, Texas	USA, Texas	Sint Maarten
5.	32	M	Puerto Rico	Puerto Rico	USA, Wisconsin
6.	31	M	USA, California	USA, California	USA, Colorado
7.	26	M	USA, Pennsylvania	USA, Pennsylvania	–
8.	24	F	USA, California	USA, California	Israel, USA, New York
9.	32	M	Germany	Germany	USA, California
10.	30	F	USA, New Jersey	USA, New Jersey	–
11.	25	M	USA, Nebraska	USA, Nebraska	–
12.	31	M	Liberia	USA, Minnesota	–

Most participants had previous educational backgrounds in science-related fields, while some had backgrounds in medical humanities, psychology, or business. Several participants also held advanced degrees or had prior professional experience in areas such as nursing, laboratory work, or military service.

Figures 1, 2 presents a summary of the key decision factors, challenges, and adaptation experiences for students pursuing medical education abroad.

**Figure 1:**
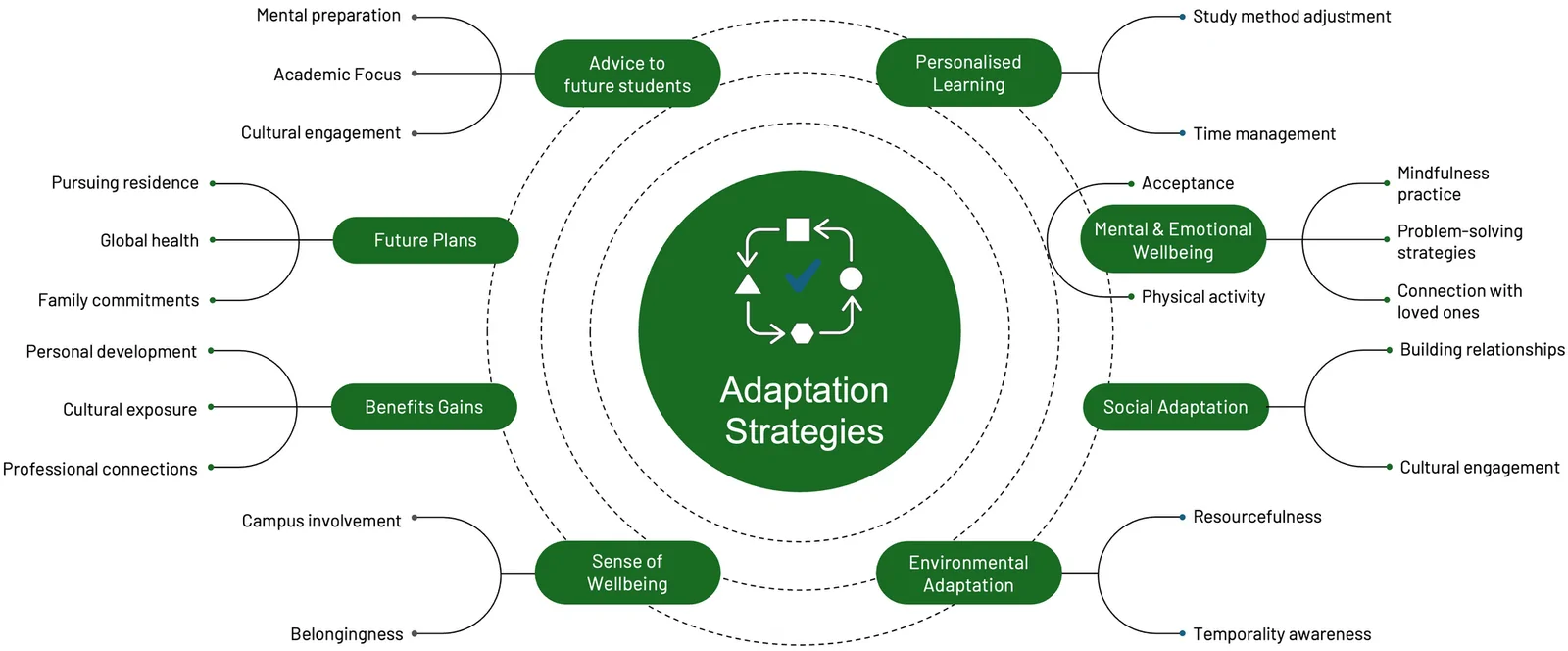
The opportunities and challenges that moderate the decision of medical students to pursue studying medicine abroad.

**Figure 2:**
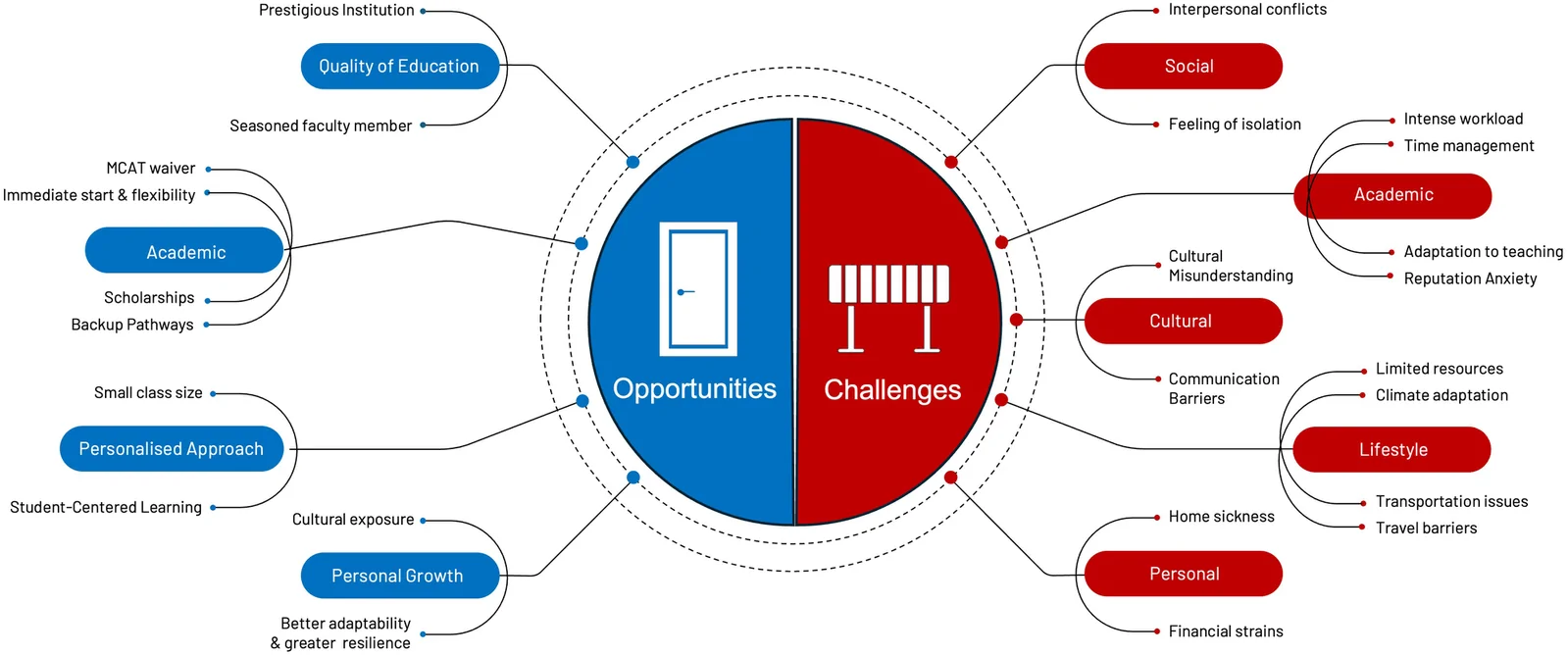
Adaptation strategies that international students reported to cope with studying medicine in different contexts.

### Opportunity-driven decision

Many students opt to attend this medical school in the Caribbean because it provides a unique opportunity to pursue their dream careers in a way that aligns with their personal educational backgrounds and financial circumstances. There's a wide range of opportunities that were indicated by participants, as discussed below with representative quotes.

1. Educational opportunities

Participants were attracted by a prompt entry into medical education, application without Medical College Admission Test (MCAT) score, and scholarships that broadened accessibility. For those who faced barriers in traditional routes to medical school, this program offered a viable alternative to pursue their medical careers.

“*Most people look at the Caribbean as a backup plan.*”

2. Quality of education

Participants highlighted the program's credibility through ties with prestigious institutions and its faculty of practicing doctors with recent USMLE experience.

“*A big thing of why I chose to consider this school was that the way it was explained to me was that it’s not a Caribbean school. It’s a U.S. school located in the Caribbean.*”

3. Personalized and supportive learning environment

A low faculty-student ratio allowed for personalized attention and a more intimate learning experience.

“*And it is much more difficult being in [big] institutions because I can be number 437 out of 500. No one knows my name or anything. Yet, here, [At ACSOM] I knew that the classes were going to be much smaller. So at least people will know my name, you know? .. And they were going to attend to my needs and I can actually work with them as well.*”

Students valued the personalized, student-centered approach and the faculty's genuine support, which fostered a positive learning environment.

“*If they believe in their students, I'm pretty sure their students will believe in them.*”

4. Personal growth

Studying in a new cultural environment appealed to those seeking to broaden their horizons. “*It only takes a very small event to shift an entire life.”*

5. Reflection on children's future education choices

Participants varied in whether they would make the same decision for their children. Many stressed *supporting their children's choices* with emotional and financial backing, while some *recommended their own school* for its supportive environment and quality education. Others favored *studying abroad* for growth and independence. A few voiced *uncertainty*, citing challenges such as residency disadvantages and the rigors of medical training, urging children to research and decide carefully.

6. Reflection on alternative plans and decision factors

Participants emphasized their *determination to pursue medicine*, saying they would retake exams, reapply, or pursue advanced degrees if not admitted to their current school. Opinions varied on whether they would still choose it over studying in the U.S., Europe, or Canada: some valued its curriculum and faculty, others preferred alternatives for residency advantages or reduced stigma, while some said the choice would depend on factors like resources, safety, or family proximity.

### Challenges during medical education abroad

Relocating to the Caribbean for medical school presented participants with a multitude of challenges that impacted various aspects of their lives. These challenges were grouped into five categories, as follows:

1. Academic challenges

Participants faced challenges with the intense workload, time management, and adapting to flipped-classroom methods, though many valued active learning and deeper understanding. Some also experienced anxiety over the stigma of attending a Caribbean medical school and its impact on residency prospects.

2. Social challenges

Participants faced challenges in a diverse cohort, including personality clashes, feelings of isolation, and experiences of perceived discrimination.

“*I learned how to still be mindful and respectful and still acknowledge people that you may not personally get along with.*”

“*People I thought I could depend on as friends here were not there for me at that time.*”

3. Lifestyle and environmental challenges

Scarcity of familiar foods and goods required lifestyle adjustments. Limited social activities were also points of frustration, with some students feeling isolated.

“*The grocery store and the selection are just super limited, right? There's not really anything you could pick from there.*”

“*We don't have a Starbucks, buddy, sorry, like, you know, it's a bit different.*”

Participants faced challenges with tropical climate adaptation, limited transportation, and costly, indirect travel home.

“*You were not able to just even go home and just see your family really quickly because, I mean, the flight travel time in total would be like 19, 20 h at least.*”

4. Personal challenges

Separation from family and missing significant life events weighed heavily on participants. “*One of the challenges is having to miss milestone events in your loved one's life.”* Participants experienced stress from financial strain, health issues, global events, such as pandemics or political unrest, and the impact of medical school on personal life, particularly in terms of relationships and family planning.

5. Cultural sensitivity and differences

Participants faced communication barriers and cultural misunderstandings, learning to adapt interactions to avoid conflicts. *“The biggest challenge was just adjusting to a new place, new culture, new people.”*

### Adaptation experience and strategies

Students' experiences vary widely, with each individual developing different strategies to overcome the challenges based on their personality. Along the way, they appreciate the benefits that help them adapt, offer valuable tips for future students, and form plans for their own future paths. International medical students reported eight strategies to cope with the new glocal ecosystem at ACSOM, as discussed below.

1. Personalized learning strategies

Participants adapted study methods and improved time management, using faculty and peer guidance to enhance learning, reduce stress, and boost performance.

“*I'm making notes and putting them around my room just so I can see them and never miss them again.*”

2. Mental and emotional well-being

Engaging in meditation and therapeutic reflection helps to manage stress.

“*I meditate before I go to bed. I kind of forgot everything that happened bad that day before. And so that way in the morning, I don't wake up with any negative feelings about what happened the day before, and I just forget. So that's really good.*”

Students managed stress through physical activity and by maintaining a positive, accepting mindset toward challenges.

“*It's hard, but yeah, I think you just take it as you go and just learn to be grateful for the opportunities that you are given and accept the hardships that it comes with.*”

Participants coped by establishing routines, using problem-solving strategies, and staying connected with loved ones to manage stress and homesickness.

3. Social Adaptation

Students adapted socially by forming supportive networks, collaborating across cultures, and learning respect for diversity. Despite occasional conflicts, they valued the growth gained from these interactions.

“*Conflicts are always going to happen. [..] So you have to learn a lot from people. You have to kind of understand different cultures and aspects of other people's lives because not everyone lives the same.*”

“*It's important to be mutually respectful to everybody and to have value and respect towards, you know, whoever is in your class, no matter like, whatever the vibes are.*”

Engaging with the local population allowed students to integrate into the community and enhance their cultural understanding, making the experience more fulfilling.

4. Environmental adaptation and practical adjustments

Participants managed food and environmental challenges by being resourceful and viewing difficulties as temporary while focusing on long-term goals.

“*I was telling myself, okay, I'm willing to take it because it's temporary.*”

5. Sense of Belonging

Participants described the campus as open, friendly, and supportive, valuing approachable faculty and a collaborative learning environment.

“*I like the environment. I like how open faculty and students are. I like how we're kind of adjusting, figuring things out as a new school. [..] Different perspectives, this whole thing is about bringing different ideas together and it's not always going to go smoothly. But it always turns out for the best.*”

Students' sense of belonging varies—some feel connected through academics, faith, or purpose, while others struggle with exclusion or uncertainty, especially when facing academic or social challenges.

6. Benefits Gained

Participants reported personal growth, resilience, and improved communication, alongside cultural exposure and meaningful relationships in a diverse, collaborative environment. “*I think it also adds to your skill set, being adaptable because, I mean, especially in this world, you have to adapt fast to new environments. And I think this is what this teaches you. Being able to live with limited resources.”*

7. Future Plans

Students aim for specialized U.S. residencies, global health opportunities, and a balance between demanding careers and personal life.

8. Advice to Future Students

Participants highlighted mental readiness, resilience, and supportive strategies as key to handling medical school challenges.

“*My advice will be to enjoy the process and enjoy every single day because sometimes we get lost planning the future and we lose track of the present because you have a lot of studying [..] So for me it will be to enjoy the process. Prepare that it is going to be ups and downs, but I think it will be about consistency. This is not like a short run. It will be a long road.” “I would say, number one, stay focused. And number two. Keep your circle small. Don't open up to everyone. Because it will throw you off course.*”

In conclusion, participants recommended early arrival, practical preparation, strong study habits, faculty engagement, and embracing local culture for a well-rounded experience.

“*Be open to different perspectives. And go out and meet people. Go out into the community, kind of hang out a bit more with people outside the school, go meet random strangers, talk to them. I think that has been more impactful than actually some talks.*”

## Discussion

The findings shed light on the dynamics and factors that contribute to the decision to study medicine abroad, with a particular focus on the Caribbean context. Participants reported key drivers, challenges, and the adaptation strategy they preferred to cope with the new context that is different from their peers who study medicine at home. We will elaborate on the above variables and present salient practical implications to guide students who may select similar trajectories.

### Motivations for studying medicine abroad

The participants in this study chose to attend the Caribbean medical school primarily because it provided a unique opportunity to pursue their dream careers in a way that aligned with their personal educational backgrounds and financial circumstances. Consistent with the modified push-pull theory, their decisions were influenced by a combination of push, pull, and conditioning factors (13). The main *push factor* was a strong commitment to a medical career and personal growth, motivating participants to pursue alternative pathways when traditional routes were limited (9, 16). The *pull factors* were more pronounced and included immediate program start dates, MCAT waivers, less competitive admissions processes compared to American schools, and the availability of scholarships. These factors made the Caribbean medical school an accessible and attractive option for students who faced barriers in traditional admission routes (18, 19, 22).

The personalized education, with small classes and faculty who knew students by name, was highly valued, contrasting with their previous experiences of anonymity in larger institutions. This supportive environment illustrates Tinto's theory that academic integration and personal attention boost student retention and success (42, 49). The focus on high-quality education, with reputable institutions and faculty who are practicing doctors with recent board experience, addressed concerns about standards and career prospects, highlighting the pull of institutional credibility (20, 21). Additionally, the opportunity for cultural exposure and personal growth through studying abroad added to the program's appeal, aligning with motivations identified in previous studies where experiencing different cultures enhances both personal and professional development (3, 4).

Conditioning factors that could hinder the decision to study abroad included financial constraints, isolation from family due to the island location, and concerns about the stigma associated with attending a Caribbean medical school. These factors required careful consideration, as they could impact students’ well-being and future career opportunities (8, 9). For some participants, attending a Caribbean medical school was viewed as a backup plan given the competitiveness of traditional admissions (4, 11). While recognizing the risks of enrolling in a new institution with limited precedent, students valued the immediate and personalized opportunities offered. Their determination to pursue medicine “no matter what” reflected resilience and dedication, key to overcoming the challenges of studying abroad. Students chose the school as the best available path to begin their medical careers, valuing accessible admissions, personalized education, and career alignment despite potential drawbacks. Their calculated risk-taking and resilience highlight the need to understand international students' motivations to design effective support systems.

### Challenges

The challenges faced by international medical students fell into two main categories: medical education and relocation to a resource-limited island nation.

*Challenges Related to Medical Education*

Participants faced significant academic, social, and personal challenges throughout their medical education. Academically, the heavy workload, fast-paced curriculum, and new teaching methods, like the flipped classroom, demanded significant adjustment, reflecting known pressures in medical education (5, 6). Socially, students encountered hurdles in navigating new environments, including building relationships with peers and faculty, managing interpersonal conflicts, and integrating into a diverse cohort, often accompanied by feelings of isolation and uncertainty, as reported in previous studies on international medical students (9, 10). On a personal level, health concerns and financial constraints further compounded their experiences, with the demands of maintaining well-being and coping with tuition fees and living expenses reflecting broader findings on the significant impact of personal and financial stressors on international students (26, 31). These challenges contributed to so-called academic dissonance, when medical students experience stress and psychological conflict when their existing cultural norms, study skills, learning habits all clash with unfamiliar expectations of their new medical school. Challenges Related to Relocation

*Relocating to a new country caused participants cultural shock, homesickness, and emotional strain, worsened by the isolation of studying on an island in a developing country. Adapting to different norms, limited resources, transportation issues, and few recreational options reduced quality of life and increased family separation, consistent with literature on cultural adaptation (8, 12, 27). Communication challenges, arising from accents, dialects, and intercultural nuances, further complicated adaptation, highlighting the importance of communication competence in adaptation as emphasized by Kim's theory (36).*

*In addition, studying at a newly established Caribbean medical school introduced reputation anxiety, with participants expressing concern that the institution's lack of alumni, networks, and established credibility might negatively affect their career prospects—an underexplored issue in existing research that highlights the psychological impact of institutional reputation (4).*

*These challenges, viewed through Berry's acculturation model (39, 50), demonstrate the compounded effects of academic, cultural, environmental, and reputational stressors, underscoring the need for an integrated model of adaptation that considers psychological, sociocultural, and logistical dimensions (40). Understanding the multifaceted nature of these experiences is essential for medical schools, which must move beyond academic support alone and implement comprehensive systems addressing cultural orientation, mental health, logistical assistance, and reassurance regarding institutional reputation to better support international students.*

### Adaptation strategies

The international medical students used diverse strategies to adapt abroad, reflecting the interplay of resilience, cultural adjustment, and coping mechanisms. According to Berry's acculturation model, participants mainly followed a **separation** strategy—maintaining ties to their home countries, planning to return for residency, and showing little interest in long-term integration into the Caribbean (35). This pattern is common among those relocating temporarily to resource-limited regions for education (51). Despite this, students developed a sense of belonging on campus, engaging with peers and faculty in a supportive environment. This partial integration reflects Berry's view that adaptation can differ across social and institutional domains (39).

Drawing on Kim's integrative communication theory, participants showed resilience and commitment, using academic adjustments, support-seeking, and openness to new experiences to enhance adaptation (36). Applying Bennett's DMIS, students moved from denial or defense toward acceptance and adaptation, gaining intercultural sensitivity (37). While few sought full cultural integration, their growing competence helped them navigate campus life academically and socially.

Tinto's student integration theory emphasizes the importance of both academic and social integration for student success (49). Supportive institutional factors, such as small classes, approachable faculty, and collaborative learning, helped students feel engaged and integrated on campus, mitigating challenges of studying abroad and supporting well-being.

The students employed both problem-focused and emotion-focused coping strategies, as described by Lazarus and Folkman's transactional coping theory (52). Problem-focused strategies involved building supportive relationships, engaging with the local community, adapting study methods, managing time, and addressing resource limitations. Emotion-focused strategies included mindfulness, exercise, routines, accepting temporary challenges, staying connected with loved ones, and keeping perspective on their time on the island as a step toward career goals.

These adaptive strategies led to better mental health and academic outcomes, reinforcing the idea that effective coping mechanisms are essential for successful adaptation (53). Students using maladaptive strategies like withdrawal faced more stress and poor adaptation. Participants highlighted the importance of mental and practical preparation, emphasizing proactive strategies for success in international medical education.

### Implications for medical institutions

Medical institutions should build comprehensive support systems for international students, addressing academic, cultural, emotional, and practical needs. This includes academic resources, study guidance, cultural orientation, accessible mental health services, and help navigating daily life in resource-limited settings. Reputation anxiety, particularly in new Caribbean schools, can be reduced through transparent communication about accreditation, affiliations, alumni success, and career support. Strengthening faculty quality, curricula, and external evaluations enhances credibility. Finally, fostering inclusivity through collaborative learning, peer networks, and cultural activities promotes integration and student well-being.

For medical students who are willing to travel to study medicine abroad, we may offer a couple of helpful tips in view of the current study. They need to explore the real motives (push and pull factors) before making such a move. It is not enough to gather information on the target country or shortlisted medical schools, but they need to study the educational ecosystem and the strategies used in teaching, learning, and assessment. They also need to consult peers who are studying there or have concluded their studies and navigate their future careers after graduation. It's important to join the *community of learners* in that context as early as they can, while they are in their home countries. Once they make the decision, international medical students need to adopt a *resilient* attitude. They should expect challenges of different kinds, as reported by participants. An insightful point to remember is that the sense of belonging and cultural competence develop gradually through engagement with peers, faculty, and the local community.

#### Phenomenology vs. Grounded Theory

The current study does not intend to create a new theory from the lived experience of international medical students, because this may need a larger sample size across different contexts and require a dynamic process of data collection and analysis simultaneously. For future studies, we recommend a grounded theory approach to offer a more comprehensive understanding of the complexity of the decision-making process of medical students to study abroad and the implications for their future careers, whether they choose to return home or practice medicine in their new context.

### Limitations and recommendations for future research

The study offers valuable insights but it is not without limitations. With only 12 participants from a single Caribbean medical school, the findings may not represent the broader population of international students, nor are they easily generalizable to other contexts. The research also reflects a specific time frame, limiting understanding of long-term adaptation. Yet, the purpose of this study was not to generalize to other contexts, but rather to report an in-depth understanding of what contributes to a *glocal context* like ACSOM of the Caribbean. We encourage scholars to use similar approaches in other contexts and explore the similarities and differences of challenges and coping strategies of international students.

Further research with larger, more diverse samples across multiple institutions and countries is needed. Longitudinal studies could capture evolving challenges and strategies, while comparative research across settings may reveal unique barriers and effective practices. Exploring issues such as reputation anxiety, resource-limited environments, and the role of new institutions could inform support systems and policy development.

An additional limitation is that all participants were in the early stages of their medical education. This reflects the context of the study, as ACSOM was newly established and had admitted its first cohort only in 2023; consequently, students with more advanced experience at the institution were not available for recruitment at the time of data collection. The findings therefore primarily capture students' decision-making, transition, and early adaptation experiences rather than their lived experiences across the full trajectory of medical education. Future longitudinal research should explore how international students' experiences, challenges, and adaptation strategies evolve as they progress through medical school.

## Conclusions

This study explored the experiences of international medical students at the newly established medical school in the Caribbean, examining their motivations, challenges, and adaptation strategies through semi-structured interviews with 12 participants. Students were primarily motivated by the opportunity to pursue medical careers without traditional admissions barriers, influenced by factors such as immediate program start dates, MCAT waivers, personalized education, and institutional reputation. They faced challenges including academic pressure, social integration, limited resources, and concerns about attending a Caribbean medical school. To cope, students developed resilience through personalized learning, mindfulness, supportive relationships, and practical adjustments to their environment. The study highlighted the importance of institutional support and comprehensive systems addressing academic, cultural, and emotional needs.

## Data Availability

The raw data supporting the conclusions of this article will be made available by the authors, without undue reservation.
